# Thrombose post traumatique de la veine sous clavière droite sans fracture de la clavicule: à propos d'un cas

**DOI:** 10.11604/pamj.2015.20.256.5728

**Published:** 2015-03-17

**Authors:** Youssef Bibiche, Nabil kanjaa

**Affiliations:** 1 Département d'Anesthésie Réanimation Centre Hospitalier Universitaire Hassan II Fes, Maroc

**Keywords:** Clavicule, traumatisme thoracique, membre supérieur, Clavicule, thoracic trauma, lower limb

## Abstract

Les thromboses veineuses profondes du membre supérieur apparaissent aujourd'hui plus fréquentes du fait de l'utilisation plus large des cathéters veineux centraux; Sa survenue après traumatisme thoracique sans fracture osseuse est exceptionnelle et ses complications mortelles. Leur diagnostic clinique est parfois difficile. Le syndrome du défilé thoraco-brachial est de diagnostic plus rare et nécessite une collaboration multidisciplinaire. La recherche d'une thrombophilie ne doit pas être systématique, ce d'autant que sa découverte ne modifie en règle pas la thérapeutique. L'enquête étiologique en présence d'une thrombose des membres supérieurs doit être rigoureuse, guidée par l'interrogatoire et l'examen clinique et en aucun cas une série systématique d'examens complémentaires ne doit être effectuée. Les auteurs rapportent le cas d'une thrombose post traumatique de la veine sous Clavière gauche sans fracture de la clavicule.

## Introduction

Les thromboses veineuses profondes (TVP) du membre supérieur, définies par l'existence d'un thrombus dans les veines sous-clavière, axillaire ou brachiale, Elle survient généralement après la pose de cathéter veineux ou de pacemaker. Son apparition suite à un traumatisme thoracique sans fracture de la clavicule est exceptionnelle. Seul un diagnostic précoce et un traitement adéquat permettent d’éviter ses complications parfois mortelles. Les thromboses veineuses profondes du membre supérieur représentaient il ya 15 ans 3,5% de l'ensemble des TVP [1], elles apparaissent aujourd'hui plus fréquentes [2] en raison du développement des techniques de diagnostique qui en permettent un diagnostic fiable et rapide [3].

## Patient et observation

**Figure 1:**
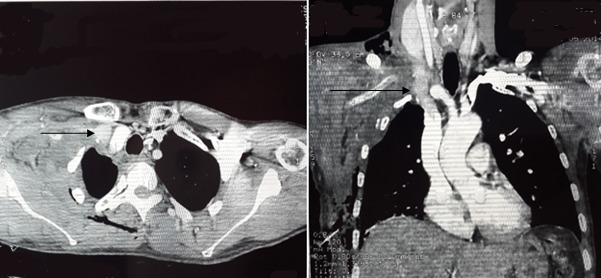
Angioscanner thoracique objectivant thrombose de la veine cave supérieure jusqu'a la veine sous-clavière droite

Patiente de 19 ans sans antécédent pathologique notable, admise au service de réanimation pour prise en charge d'un traumatisme thoracique suite à un accident de la voie publique (collision entre deux motos). Le bilan lésionnel avait objectivé un hémothorax droit avec fracture d´humérus, sans fracture des cotes ou la clavicule. Il n´y avait pas de signe de détresse respiratoire ou hémodynamique, un drainage thoracique a été réalisé associé à une analgésie multimodale. Accoure de son hospitalisation le patient a présenté des signes isthmiques de membre supérieur droit d'où la réalisation un angio-scanner thoracique objectivant thrombose de la veine cave supérieure jusqu'a la veine sous-clavière droite (Figure 1). La patiente mise sous anticoagulant après amputation du membre supérieure.

## Discussion

Les TVP du membre supérieur sont rares et représentent 2 à 4% de l'ensemble des TVP [1]. Leur fréquence augmente cependant depuis l'utilisation croissante des cathéters veineux centraux [2]. Les autres étiologies locales sont représentées par les sondes de pacemaker, les compressions tumorales et le syn-drome de défilé thoracobrachial [3, 4]; les causes générales sont essentiellement les thrombophilies, les syndromes paranéoplasique et les maladies systémiques [5, 6]. La survenue d'une TVP suite à une fracture du membre supérieur est exceptionnelle. Seuls quelques cas sporadiques ont été rapportés dans la littérature [7, 8]. Cliniquement, les TVP du membre supérieur se manifestent par: un œdème qui débute au niveau de la main puis s’étend à l'avant-bras et au bras; une douleur; une circulation collatérale cutanée; un comblement du creux sus-claviculaire. La palpation du creux axillaire peut percevoir un cordon douloureux [9]. Angio-scanner précise le diagnostic et l'extension de la thrombose. Le traitement est basé sur les anticoagulants et sur l'exérèse d'une éventuelle cause: ablation du cathéter ou de la chambre implantable, chirurgie d'une côte cervicale, etc. Les TVP des membres supérieurs étaient classiquement considérées comme peu emboligènes et de bon pronostic. En réalité, elles peuvent se compliquer d'embolie pulmonaire dans 9 à 36% des cas [10].

Les autres complications sont représentées par le syndrome cave supérieur (suite à l'extension du thrombus aux veines jugulaire interne et cave supérieure) et le syndrome post-phlébitique. Chez notre patient, le mécanisme le plus probable de survenue de la thrombose est la stase sanguine conséquente d'un gène du retour veineux au niveau du membre supérieur droit. L'hypothèse de lésion vasculaire est aussi à discuter surtout qu'il s'agissait d'un traumatisme mais l'examen clinique n'a trouvé ni de foyer de contusion évident, ni d'ecchymose en regard du point d'impact. L’état d'hypercoagulabilité a été exclu par un bilan négatif de thrombophilie. Dans ce contexte traumatique, le phénomène de stase vasculaire peut être due: d'une compression de la veine sous-clavière par un hématome post-traumatique de la région. Cette éventualité a été éliminée parangioscanere thoracique; d'une phlébite d'effort appelée encore syndrome de Paget-Schroetter. C'est un syndrome propre au sujet jeune sportif en rapport étroit avec l'anatomie statique et dynamique du défilé thoracobrachial [11].

## Conclusion

La thrombose veineuse profonde est une complication exceptionnelle des fractures de la clavicule. Au vu de ses conséquences dramatiques, il faut prévenir sa survenue en évitant les contentions trop serrées, préférer les écharpes aux anneaux claviculaires en huit et discuter le traitement chirurgical surtout chez le sujet jeune sportif.
